# Pneumoperitoneum, Retropneumoperitoneum, Pneumomediastinum, and Diffuse Subcutaneous Emphysema following Diagnostic Colonoscopy

**DOI:** 10.1155/2012/108791

**Published:** 2012-09-16

**Authors:** Evangelos Falidas, Georgios Anyfantakis, Konstantinos Vlachos, Christina Goudeli, Boutzouvis Stavros, Constantinos Villias

**Affiliations:** ^1^First Department of General Surgery, 417 NIMTS, Veterans Hospital of Athens, 10-12 Monis Petraki Street, 11521 Athens, Greece; ^2^Department of Radiology, 417 NIMTS, Veterans Hospital of Athens, 10-12 Monis Petraki Street, 11521 Athens, Greece

## Abstract

Colonoscopy is a widely used diagnostic and curative procedure. Extraperitoneal perforation with pneumoretroperitoneum, pneumomediastinum and subcutaneous emphysema combined with intraperitoneal perforation is an extremely rare complication. We report a case of a 78-year-old woman presented to the emergency department with abdominal pain and diffuse abdominal, chest, neck, and facial swelling appeared after a diagnostic colonoscopy. Diagnostic and therapeutic modalities are discussed.

## 1. Introduction

 Colonoscopy is a widely used diagnostic and curative procedure. However, bleeding, perforation, and postpolypectomy coagulation syndrome may variably occur. The incidence of colonic perforation after colonoscopy is very rare and estimated in 0.19–0.21% [1]. Intraperitoneal perforation is common whereas extraperitoneal perforation with pneumoretroperitoneum, pneumomediastinum, and subcutaneous emphysema is extremely rare [2]. We report a case of a 78-year-old woman presented to the emergency department with abdominal pain and diffuse abdominal, chest, neck, and facial swelling appeared after diagnostic colonoscopy elsewhere performed 3 hours ago. 

## 2. Case Presentation

**Figure 1:**
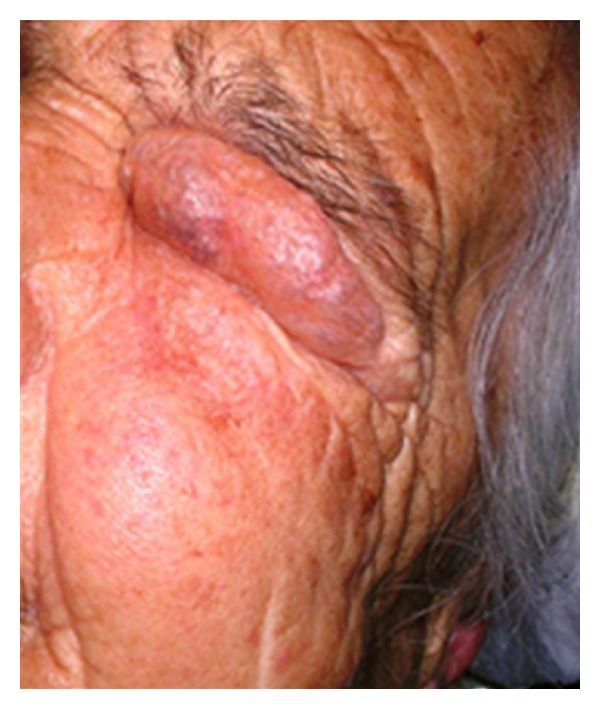
Extensive subcutaneous emphysema involving left hemiface and left orbit.

**Figure 2:**
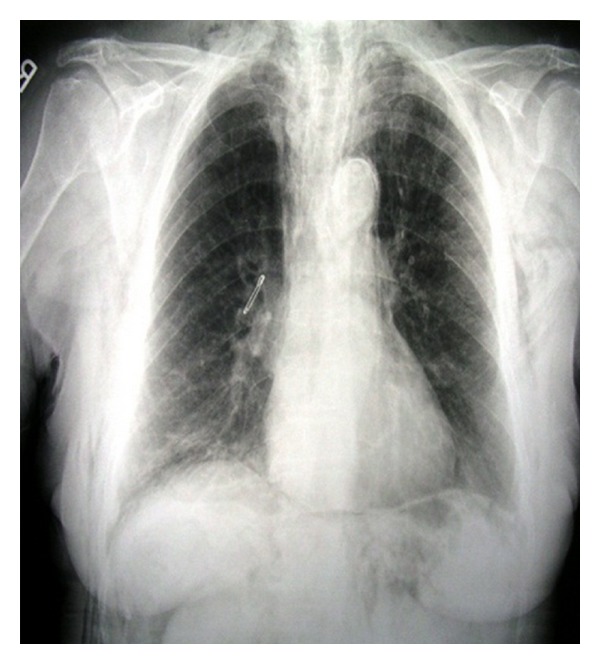
Chest X-ray demonstrating diffuse subcutaneous emphysema, pneumomediastinum, and subdiaphragmatic free air.

**Figure 3:**
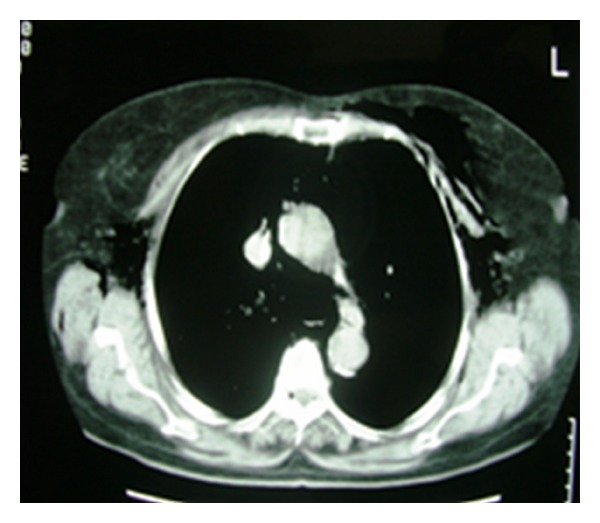
Transverse CT image of the chest revealing bilateral diffuse subcutaneous emphysema and pneumomediastinum. Free air is noted at the anatomic region of the aortic arch and the descending thoracic aorta.

**Figure 4:**
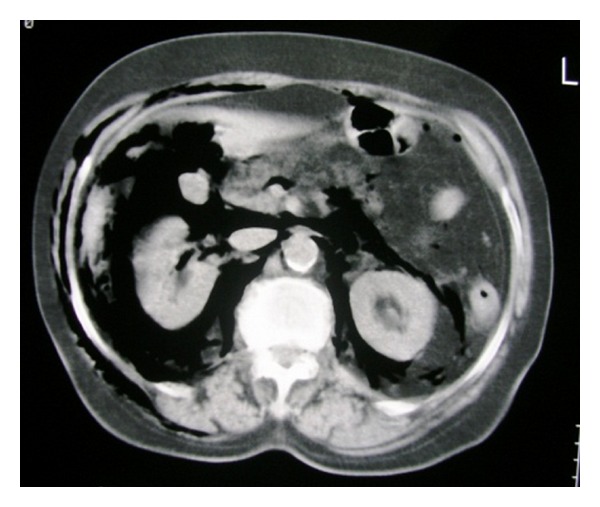
Transverse CT image of the upper abdomen demonstrating subcutaneous emphysema and pneumoperitoneum. Free air surrounds both kidneys and overlaps both great vessels.

**Figure 5:**
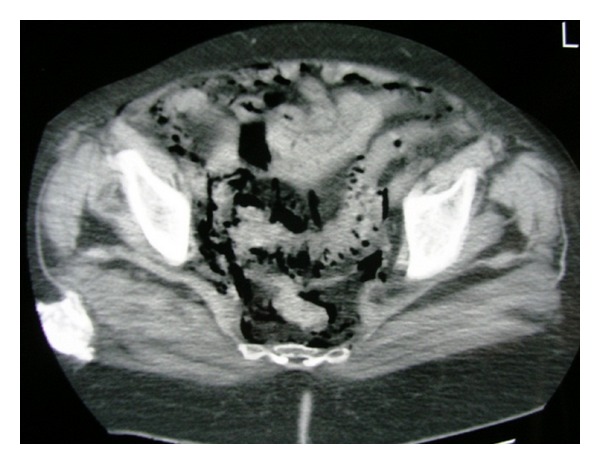
Excessive amount of free air surrounding the sigmoid colon and diverticulosis.

 A 78-year-old woman arrived to the emergency department complaining of low abdominal pain and swelling of the neck, face, and left orbit (Figure 1). She mentioned a diagnostic colonoscopy performed 3 hours ago in a private medical studio in order to investigate anemia. During colonoscopy, the gastroenterologist observed diffuse subcutaneous emphysema of the neck, face, and left orbit. He interrupted the procedure and suggested immediate transfer to our institution. Upon examination, left orbit, neck, and abdomen emphysema with crepitus were noted. Abdominal pain was mainly located to the left iliac fossa. Abnormal laboratory findings included leukocytosis (14.000/mm^3^), anemia (Hb: 9 g/dL), and minimally elevated C-reactive protein (2.32 mg/dL). Temperature was slightly elevated (37.8°C). Vital parameters were within normal limits and no signs of respiratory distress were observed (oxygen saturation 97%). Additional information was obtained from the gastroenterologist who performed the colonoscopy. He mentioned extensive diverticular disease of the sigmoid colon and insufficient mechanical preparation of the colon with solid stools. He attributed the perforation to the effect of the air insufflations in connection with the observed diverticula. The patient underwent chest radiograph and chest and abdominal computed tomography (CT) scan. Right subdiaphragmatic air and diffuse subcutaneous emphysema were observed (Figure 2). The CT scan described pneumoperitoneum, retropneumoperitoneum, and pneumomediastinum as well as diverticulosis of the sigmoid colon (Figures 3, 4, and 5).

 We initially decided a conservative treatment. Fluids and antibiotics (ciprofloxacin and metronidazole) were administered. 24 hours later, the patient's clinical condition was worsening. Temperature was elevated (38.8°C) while additional laboratory findings revealed leukocytosis (22.000/mm^3^) and elevated C-reactive protein (13,5 mg/dL). Clinical examination revealed intense abdominal pain with rebound tenderness.

 Surgery was decided in view of the clinical findings. Laparoscopy was not available by that time. Upon laparotomy, diffuse diverticula of the sigmoid colon were observed. The epiploic appendices, the mesocolon, and the retroperitoneum were full of air as well as the greater omentum. The point of perforation was identified near a diverticulum. Diffuse diverticula of the sigmoid colon were also found. Thickness and local inflammation were limited. A small amount of peritoneal fluid and stools were observed in the peritoneal cavity. Segmental resection was performed followed by an end-to-end anastomosis. Postoperative course was uneventful and she was discharged 10 days after the initial observation. 

## 3. Discussion

 Colonoscopy is a common and safe diagnostic and curative procedure. Complications such bleeding, perforation, and postpolypectomy coagulation syndrome may occur. Perforation incidence after colonoscopy ranges from 0.19 to 0,21% and usually happens after therapeutic procedures (0.2–0.44%). This complication rarely occurs after diagnostic colonoscopy (0.06–0.17%) [1]. Sigmoid colon is frequently involved [2].

 Perforation may be caused by different mechanisms. Pneumatic perforation is the consequence of high pressure insufflations resulting in excessive distension of the bowel wall and rupture. Mechanical rupture should be attributed to the pressure performed by the endoscope against the colonic wall. Perforation commonly occurs at points of previous colonic lesions or weakness such as diverticula with or without diverticulitis, inflammatory, or neoplastic processes and recent colonic operations [3]. Forcible herniation of the mucosa may occur during air insufflations. This makes the mucosa permeable to the air without an evident point of perforation [4]. Thermal injury of the colonic wall due to high power of current during electrocoagulation for polypectomies is an additional factor of perforation [1, 3]. Advanced age, comorbidities, and endoscopic skills of the operators further influence the risk of this complication [2].

 The retroperitoneal perforations are uncommon. Cirt et al. [1] reviewed the literature from 1974 to 2006 and found 24 reported cases of retroperitoneal perforation with various clinical presentations. Among them, fourteen cases were associated with polypectomies while only two were surgically treated. Regarding the mechanism of air diffusion, Maunder et al. [5] divided the soft compartment of the abdomen, chest, and neck into four interconnected spaces: (1) subcutaneous tissue, (2) prevertebral tissue, (3) visceral space, and (4) perivisceral space. Air insufflations in one space may pass into the others. Perforation favors the air passage into the retroperitoneum. The air diffuses along fascial planes and large vessels and through the diaphragmatic hiatus, occupies the mediastinum, and spreads to the neck [5]. Pneumothorax, following colonic rupture due to colonoscopy, has been rarely reported and its development could be attributed to air decompression into the pleural cavity or to extension of pneumoperitoneum to the pleura through small diaphragmatic fenestrations [4, 6]. 

 On the other hand, combined intra- and extraperitoneal paerforation during colonoscopy is extremely rare. In the study of Cirt et al. [1], only eleven cases were identified in the literature. There is not an ideal treatment of similar cases and the choice is commonly based on a case-by-case basis. However, it is commonly accepted that a conservative treatment could be achieved in patients in good general condition, in clinical and laboratory absence of peritonitis signs, hemodynamic stability, and sufficient colonic mechanical cleaning [1]. All patients under nonoperative management should be closely monitored while the clinical condition should improve within 24–48 h. Success rate of non-operative management is estimated from 33% to 73% [7]. Endoscopic clipping followed by conservative treatment, has been recently reported and could be a valid approach in patients with small lesions and without signs of peritonitis [3]. Surgical treatment is indicated when there is evidence of fecal content into the bowel during colonoscopy and of peritonitis signs. Additional elements such as the presence of distal obstruction to the perforation site, the absence of clinical improvement or the worsening after conservative treatment, and the concomitant colonic morbidities further enforce the surgical option [1, 7]. Simple closure with sutures depends on the delay of diagnosis, size of the perforation and quality of the damaged colonic wall. Colostomy, segmental resection, and Hartmann's procedure are valid approaches commonly related with the age of the patient, timing in diagnosis, co-morbidities and degree of peritonitis [1, 7].

 In our case, the patient had a simultaneous intra- and extraperitoneal perforation. Diagnosis of retroperitoneal involvement was posted in relation to the subcutaneous emphysema. Intraperitoneal perforation was clinically suspected and confirmed through radiologic examination. We initially opted for the non-operative management. The important changes of the patient's clinical condition with evident signs of peritonitis made us consider operation in the means of infection source control. Minimal fecal contamination and timing in diagnosis (less than 48 h from the initial observation) allowed us to perform resection with anastomosis.

## 4. Conclusion

 Intra- and extraperitoneal colonic perforation following diagnostic colonoscopy is extremely rare. Non-operative management may be achieved when no signs of peritonitis or hemodynamic instability exist. The choice of operative management should be strongly related with clinical, laboratory, and radiologic changes during non-operative management, with diffuse peritonitis and with colonic pathology that requires surgery.
